# Bronchoscopy and interventional radiology: a strategic alliance in thoracic disease management

**DOI:** 10.36416/1806-3756/e20250207

**Published:** 2025-09-22

**Authors:** Bianca Fidelix Espindula, Iunis Suzuki, Altair da Silva Costa, Priscila Mina Falsarella, Rodrigo Gobbo Garcia

**Affiliations:** 1. Centro de Medicina Intervencionista, Hospital Israelita Albert Einstein, São Paulo (SP) Brasil.

The Latin word *mederi*, which means to heal or to treat, originated the term *medicus*, used in Ancient Rome to describe those who cared for human health through observation and empirical methods. Interestingly, *mederi* can also be translated as “to choose the best path,” a definition that metaphorically anticipates the modern interdisciplinary cooperation among medical specialties, an approach potentially capable of defining the most appropriate diagnostic and therapeutic route for each patient. 

Medicine has witnessed significant evolution in recent decades, marked by the advent and rapid expansion of minimally invasive specialties. With the exponential growth of knowledge, specialization has become inevitable. The volume of information and the sophistication of diagnostic and therapeutic tools have surpassed the capacity of any single professional to master them all. Traditionally, hospitals organize their departments on the basis of technological or operational affinity, not always prioritizing the clinical journey of patients. This dynamic has led to the grouping of services based on structural convenience: respiratory endoscopy is integrated with digestive endoscopy because of equipment similarity, and interventional radiology is placed within imaging departments because of shared infrastructure. However, such an organization rarely aligns with the best clinical interest of patients. 

In response to this structural mismatch, our institution has adopted an innovative model in which bronchoscopy and interventional radiology share the same department and physical space. Although at first glance these might seem like incompatible fields because of differences in instruments, workflows, and team training, experience has shown that this strategic partnership, by placing the patient at the center of decision-making, promotes more accurate and personalized clinical approaches. Daily collaboration and physical proximity facilitate real-time discussion of cases, an especially valuable interaction in complex clinical scenarios in which isolated approaches often fail to provide satisfactory diagnostic or therapeutic solutions. 

Bronchoscopy and interventional radiology are complementary pillars in the investigation of mediastinal, hilar, and pulmonary lesions. Both disciplines share the fundamental goal of providing effective and less morbid alternatives to traditional surgical procedures. In many cases, bronchoscopy may rely on interventional radiology to reach a diagnostic or therapeutic target, and, conversely, interventional radiology can benefit from the expertise of bronchoscopy in airway management or for complementary diagnostic approaches. 

Real-time interdisciplinary dialogue helps identify the best access route to challenging targets, evaluating, for example, whether advanced bronchoscopy, combining radial EBUS, fluoroscopy, or cone-beam CT, offers advantages over CT-guided transthoracic biopsy (Figure 1). This decision becomes especially relevant in patients at high risk for pneumothorax, such as those with emphysema, those with pulmonary fibrosis, and those with a single lung, or when nodules are located deep within the lung parenchyma. Similarly, for mediastinal lesions, the choice between EBUS-TBNA and CT-guided transthoracic biopsy is carefully considered, with lesion location, proximity to vital structures, and patient risk profile being taken into consideration. The goal remains the same: to maximize diagnostic yield while minimizing morbidity.^1^ ^,^ ^2^

**Figure 1:**
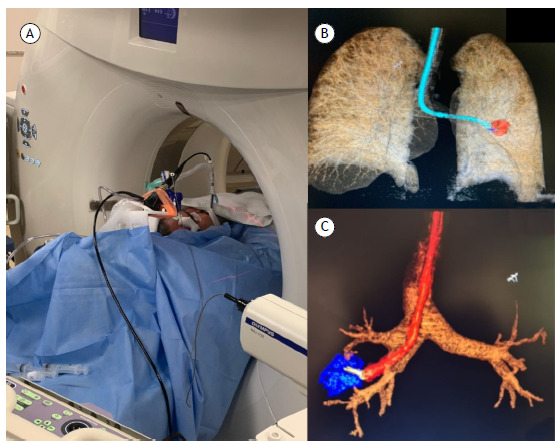
Combination of bronchoscopy techniques using radial EBUS and CT for the diagnosis of a pulmonary nodule. In A, patient in the procedure room, undergoing simultaneous bronchoscopy with radial EBUS and CT. In B and C, three-dimensional CT reconstruction to guide the biopsy procedure.

The integration of bronchoscopy and interventional radiology also brings significant logistical benefits for the patient. By combining these specialties in a shared environment, diagnostic tests and staging procedures can be performed during a single anesthetic session, a principle known as the one-stop shop. This approach significantly reduces the time to reach a definitive diagnosis; prevents repeated hospital admissions and multiple anesthetic exposures; and accelerates treatment initiation. 

In addition to diagnosis, airway management during interventional procedures represents another point of convergence between these specialties. Bronchoscopy allows intubation of difficult airways and placement of double-lumen tubes during percutaneous interventions (Figure 2). It also plays a role in managing hemoptysis resulting from transthoracic biopsies, assisting with the placement of endobronchial blockers and the application of other hemostatic measures, thus ensuring rapid and effective bleeding control. In turn, interventional radiology promptly detects and treats pneumothorax following transbronchial biopsy by performing image-guided chest tube drainage. This synergy and immediate response enhance safety standards and ensure timely management of adverse events.^3^

**Figure 2:**
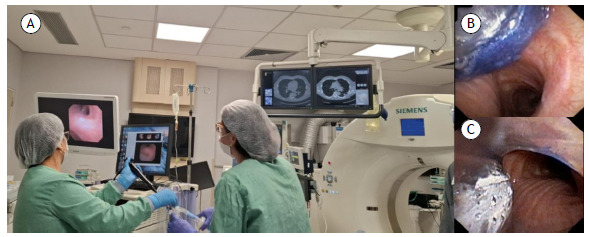
Placement of a double-lumen endotracheal tube for a CT-guided biopsy of a peripheral nodule with a high risk of bleeding. In A, patient undergoing intubation in the procedure room. In B and C, bronchoscopic view of the intubation procedure with a double-lumen endotracheal tube.

Although differences in workflow, team training, and equipment logistics may pose initial challenges, the incorporation of bronchoscopy into interventional radiology units or broader interventional medicine departments has shown clear benefits. Shared protocols, joint training, and integrated procedure rooms foster a culture of safety, improve outcomes, and accelerate the learning curve for all professionals involved. This promotes an optimized approach with interdisciplinary discussions focused on selecting the best diagnostic and therapeutic strategy for each patient.^4^

Our experience illustrates a fundamental transformation in modern medicine: the shift from a fragmented, infrastructure-centered model to a patient-centered approach supported by multidisciplinary collaboration, diagnostic optimization, and risk mitigation. This redirection not only improves clinical outcomes but also promotes innovation, strengthens professional training, and drives the development of new therapeutic strategies. 

The traditional separation between bronchoscopy and interventional radiology, historically driven by logistical convenience, no longer meets current demands. Integrating these practices has proven to be feasible, safe, and, above all, superior in addressing the increasingly complex landscape of thoracic diseases, in which diagnostic and therapeutic approaches transcend the boundaries of a single specialty. We invite the medical community to consider this collaborative model as a paradigm for the future of interventional pulmonary medicine.

We would like to highlight that the activities described in this editorial were performed at the *Hospital Israelita Albert Einstein* Center for Interventional Medicine, located in the city of São Paulo, Brazil. 

## References

[1] Green DB, Groner LK, Lee JJ, Shin J, Broncano J, Vargas D (2021). Overview of interventional pulmonology for radiologists. Radiographics.

[2] Wahidi MM, Herth FJF, Chen A, Cheng G, Yarmus L (2020). State of the Art Interventional Pulmonology. Chest.

[3] Wayne MT, Prescott HC, De Cardenas J (2021). Invited commentary Better together-interventional pulmonology and thoracic radiology. Radiographics.

[4] Gesthalter YB, Channick CL (2024). Interventional Pulmonology Extending the Breadth of Thoracic Care. Annu Rev Med.

